# Strong or Weak Handgrip? Normative Reference Values for the German Population across the Life Course Stratified by Sex, Age, and Body Height

**DOI:** 10.1371/journal.pone.0163917

**Published:** 2016-10-04

**Authors:** Nadia Steiber

**Affiliations:** 1 Wittgenstein Centre for Demography and Global Human Capital (IIASA, VID/ÖAW, WU), International Institute for Applied Systems Analysis (IIASA), Laxenburg, Austria; 2 Department of Economic Sociology, University of Vienna, Vienna, Austria; Medizinische Universitat Innsbruck, AUSTRIA

## Abstract

Handgrip strength is an important biomarker of healthy ageing and a powerful predictor of future morbidity and mortality both in younger and older populations. Therefore, the measurement of handgrip strength is increasingly used as a simple but efficient screening tool for health vulnerability. This study presents *normative reference values* for handgrip strength in Germany for use in research and clinical practice. It is the first study to provide normative data across the life course that is stratified by sex, age, and body height. The study used a nationally representative sample of test participants ages 17–90. It was based on pooled data from five waves of the German Socio-Economic Panel (2006–2014) and involved a total of 11,790 persons living in Germany (providing 25,285 observations). Handgrip strength was measured with a *Smedley* dynamometer. Results showed that peak mean values of handgrip strength are reached in men’s and women’s 30s and 40s after which handgrip strength declines in linear fashion with age. Following published recommendations, the study used a cut-off at 2 SD below the sex-specific peak mean value across the life course to define a ‘*weak grip’*. Less than 10% of women and men aged 65–69 were classified as weak according to this definition, shares increasing to about half of the population aged 80–90. Based on survival analysis that linked handgrip strength to a relevant outcome, however, a ‘*critically weak grip’* that warrants further examination was estimated to commence already at 1 SD below the group-specific mean value.

## Introduction

The strength of a person’s handgrip measured with a dynamometer has come to be widely recommended as a simple but valid measure of overall muscle strength [1] and a central marker for the onset of *sarcopenia* [2], i.e., the age-associated reduction of muscle function and strength from age 50 onwards [3]. Low handgrip strength (abbreviation: 'HGS' in the following) tends to be associated with functional limitations and it is a powerful predictor of future disability, physical health problems, and cognitive decline [4–6]. Low HGS was in fact found to be a better predictor of mortality than chronological age and systolic blood pressure [7–9]. For these reasons, gerontologists have suggested to measure HGS in clinical practice to allow for an early detection of a decline in muscle mass associated with morbidity and mortality risks [9,10]. The measurement of HGS is a prime candidate for use in routine medical exams given the simplicity and low cost at which it helps to assess patients’ muscular fitness [2].

The measurement of HGS has been proposed as a key component of frailty phenotypes and it was also suggested as a central *biomarker of healthy ageing* [7]. For these reasons, HGS is measured in a great number of ‘ageing studies’ such as the US Health and Retirement Study (HRS), the Survey of Health, Ageing, and Retirement in Europe (SHARE), and the English Longitudinal Survey of Ageing (ELSA). The fact that most surveys that include measures of HGS only cover the population aged 50 and over limits life course research on HGS. Studies show that health-related behaviours such as physical activity in mid-life predict HGS at older ages [11]. Moreover, HGS in early old age (ages 56–68) has been shown to be associated with the probability of extreme longevity [12]. Much less is known about levels of HGS earlier in life and its implications for future outcomes. There is some evidence that low muscle strength among school-aged youth is associated with cardiovascular and metabolic risk factors [13], supporting the view that routine screening of muscle strength should not be restricted to older ages and that reference values across the life course are needed.

The aim of this study is to provide *normative reference values* for HGS that allow for a comparison of HGS measurements in the clinical context and in other scientific studies with values that can be considered *normal* at certain ages. The reference values are measured from a healthy reference population. Since HGS measurements show a great deal of variation across geographical regions and national contexts [14–16], it is important to have region-specific reference values. In contrast to much of the previous work that tended to be based on small and/or non-representative convenience samples, this study is based on a large random sample of test participants that is nationally representative. The presented analyses are based on pooled data from five survey waves of the German Socio-Economic Panel (2006–2014) that provide more than 25 thousand measurements of HGS from test participants ages 17–90.

This study is among the first to provide normative data across the life course (for young, middle-aged, and older adults)—the very first studies providing life course data have recently been presented by Dodds *et al*. [17] for the British context and by Peterson and Krishnan [18] for the US (see also [19]). This study is the first to provide such reference values for Germany and it is the first to provide reference values across the life course that are not only stratified by sex and age but also for *body height*. Prior studies have in part normalized grip strength for body height or body weight [18], yet in most cases normative reference values are not presented for different population groups defined along such anthropometric measures. However, given the substantial share of the variance in HGS explained by body height (over and above sex and age), it is clearly important to stratify normative reference values for participants’ body height [20].

## Prior Work

A descriptive review of available studies providing reference values for HGS measurement shows that most of them draw on small, non-representative convenience samples of test participants (see S1 Table). Only three prior studies have presented *nationally representative* reference values covering the whole *life course*–one for the British context [17] and two for the US American context ([18,19], both using the same source of data). The studies provide similar results, showing that among men peak mean values of HGS of around 49–52 kg are reached in the fourth decade of life, whereas women reach their peak value of about 31 kg on average in the third or fourth decade of life. In the seventh decade of life, mean values drop to around 41–42 kg among men and to 25–26 kg among women (see Panel A of S1 Table).

Other studies presenting reference values for broad age ranges (covering larger parts of the life course, see Panel B of S1 Table) are typically based on small samples that are not representative of the country’s population. Many of the studies draw on convenience samples recruited from various locations such as hospitals, sports clubs, universities, senior residences, or shopping malls [21–25]. Moreover, all of these studies are restricted to small sets of test participants at each given sex and age (see also [26]). Other studies are based on regional data [21,22,27–29] or on data collected in a diverse set of countries [30]. Despite methodological limitations, these studies do not tend to show radically different results compared to those from the nationally representative studies (cf. Panel A of S1 Table). Based on a convenience sample of 720 participants collected in the US, Peters and colleagues [24] show peak mean values of 49 kg for men and 29 kg for women, values dropping in the seventh decade of life to 43 kg for men and 25 kg for women. These results are remarkably close to those reported by Perna et al. [19] drawing on the representative National Health and Nutrition Examination Study (NHANES). Reference values from the North West Adelaide Health Study [29] and from a Health Survey in Rio de Janeiro [28] show somewhat lower values for the respective regions of Australia and Brazil (peak mean values of 47 kg for men in both studies; a decline in men’s seventh decade of life to 40 kg in Adelaide and to 37 kg in Rio). Comparatively higher peak values are shown by studies drawing on convenience samples from German-speaking regions and Norway (e.g., peak mean values of 54 kg for Bavarian men, 56 kg for German-speaking Swiss men, and 58 kg for Norwegian men). The values for the seventh decade of life in these studies were also somewhat higher than those reported by the nationally representative studies for Britain and the US, i.e., dropping to 45 kg for Bavarian men, 43 kg for German-speaking Swiss and Norwegian men [22,23,25]. Finally, a study of two cities near Madrid, Spain, suggests that mean values drop to 38 kg among men in their 60s. Such national comparisons appear to corroborate prior research showing that handgrip tends to be stronger in Northern and Continental European countries than in Southern Europe [15]. More robust conclusions can only be drawn based on nationally representative data. Studies discussed in Panel B of S1 Table generally provide insufficient sample sizes for subgroups defined by sex and age and only allow for tentative conclusions.

Compared to the studies presented in Panel B, studies that do *not* provide a life course perspective but that are based on sufficient sample sizes for older age-groups (see Panel C of S1 Table) are of greater value for robust inter-study comparisons. A medium-scale study of the community-dwelling Japanese population aged 60 and above [31] estimates an average HGS of 38 kg for men aged 65 and of 32 kg for men aged 75, and thus values that are somewhat lower than those reported in studies of Caucasian populations living in Britain, the US, or Northern Europe (for regional studies on Japan see also [32,33]). A study on the Swedish province of Uppsala, for instance, suggests an average HGS of 41 kg for men aged 74–76. The commonly found lower HGS among Asian compared to Caucasian men, however, does not appear to extend to women. Aoyagi and colleagues [34] compare Japanese women living in Japan aged 65–69 with Japanese and Caucasian women living in the US in the same age group. They find the comparatively highest mean HGS in the native Japanese sample.

Finally, three studies could be identified that provide reference values for certain population groups that are not only stratified by age and sex but also by *body height* (see Panel D in S1 Table). None of these studies provide reference values across the life course. The largest of these studies presents values for the United Kingdom, drawing on data from the UK Biobank, and covers the age range 39–73 [20]. The second study covers the Danish population aged 45 and above, using data from three nation-wide population-based surveys [35]. And the third one draws on data representative of the community-dwelling Irish population aged 50–85 [36]. The studies corroborate the view that it is important to account for the height of test participants when evaluating their HGS against some reference value. The Irish study suggests, for instance, that men aged 65 have an average HGS of 34 kg if they are less tall than 173 cm and of 38 kg if taller. The British and the Danish studies were able to differentiate more than two height groups, showing that average HGS increases by 2–4 kg for each 10 cm of body height (with variations in the relation between HGS and body height by age).

To sum up, published normative data for HGS are available from many countries. Typically, reference values are provided for different age and sex subgroups, whereas only a handful of studies provide reference values for subgroups defined also along the lines of *body height*. The majority of available studies furthermore pertain to a limited age range, whereas only a small set of studies provide reference values for comprehensive age ranges that cover a large part of the *life course*. The few studies taking a life course perspective report mean or median values of HGS for different sex and age groups and show a peak of HGS in the fourth decade of life followed by a gradual decline in HGS with age—for both sexes. Although prior evidence suggests that normative values would need to be stratified not only for age and sex but also for body height [20,36], to date none of the available life course studies provides reference values for height subgroups. This is the aim of the present study, i.e., to provide reference values for a comprehensive age range (17–90) and based on sufficient samples sizes for each age group and sex, to provide values for height subgroups. Many of the available studies (but by far not all) exclude persons with health limitations such as arthritis, heart conditions, inflammatory or neurological diseases. There is, however, no standard procedure used to construct a *healthy reference population*. At all events, all of the available studies are naturally restricted to test participants who are in a state of health that allows them to take part in the study and to have their HGS measured with a dynamometer. This typically excludes the institutionalized population. The average health among test participants will thus be somewhat better compared to the general population.

## Data and Methods

### German Socio-Economic Panel

This study uses anonymized secondary data, collected by the German Institute for Economic Research (DIW). The data derive from the German Socio-Economic Panel (SOEP), a household panel study providing representative data for the German population since 1984. The SOEP is approved as being in accordance with the standards of the Federal Republic of Germany for lawful data protection. The survey ethics are monitored by an independent advisory board at the DIW. SOEP data are available free of charge as scientific use files.

In 2006 when HGS was first measured, a *random* subsample of 5,528 individuals out of 32,304 survey respondents was selected to be assessed for HGS. In 2008, the measure was repeated for the majority of the 2006 test participants (longitudinal stability of 76%) and from 1,437 individuals HGS was measured for the first time in 2008 [37]. HGS was again measured in 2010, 2012, and 2014; including both repeat testing (longitudinal sample providing HGS measures at more than one age) and testing on refresher samples measured for the first time in these years. The HGS measurement included more than 5,000 participants in each of the four measurement years (annual response rates ranging between 95% and 97%).

### Sample

The sample is restricted to participants ages 17–90 (due to small sample sizes outside this age range). For details on sample sizes in the chosen age range in the five measurement years, see S2 Table. In terms of anthropometric measures, the sample is restricted to men who are between 160 and 200 centimetres tall and to women with a body height between 150 and 184 centimetres (i.e., excluding 1% of participants). Moreover, it excludes 222 outliers, identified from sex-specific regressions that model HGS as a function of age and body height in linear and quadratic form. Those with standardized residuals of above +/-3 SD are removed from the sample (0.8% of participants). In the aim to provide results for a *healthy reference population*, the sample is restricted to those able to participate in the HGS test and attaining a value of at least 10 kg. This threshold can easily be met by all reasonably healthy persons even at higher ages (e.g., less than 2% of participants aged 80–90 attain values below 10 kg). Overall, the lower bound on the HGS test result of 10 kg excludes 0.09% of measurements. Finally, the sample excludes those who score in the lowest 5% of the Physical Component Summary Scale (PCS) of the SF-12 module in the SOEP which measures functional health and well-being based on twelve questions. The SF-12 is considered a quasi-objective measure of health [38,39]. It covers eight health domains that are summarized in two dimensions: physical health (PCS) and mental health (MCS). The PCS accounts for physical functioning, role limitations due to physical health problems, bodily pain, and general health perceptions. The PCS score is z-standardized to a mean value of 50 and a SD of 10 (the cut-off value to define the lowest 5% of the PCS is 30). The final sample for analysis involves 25,285 observations.

### Handgrip strength

Handgrip strength in kilogrammes is measured with the *Smedley S DynamometerTMM Tokio* 100kg. Prior research suggests that different dynamometer types and brands produce similar results, i.e., reference values are robust to the dynamometer type used [17], and that values taken with a Smedley dynamometer very strongly correlate with those taken with the commonly used *Jamar* dynamometer [40]. The examination procedure in SOEP foresees that two measures of HGS are taken from each hand. Following published recommendations [1,37], the *maximum* value achieved with either hand is used as a summary measure of a person’s isometric strength of the hand and forearm muscles. This is a common choice of summary measure in prior research (cf. S1 Table), yet it is worth mentioning that studies show similar results irrespective of whether they use average or maximum values achieved in multiple trials [41].

### Statistical analysis

For the statistical analyses, data from five waves of the SOEP are pooled, resulting in 25,285 HGS measurements (from 11,790 persons). Following [17], all available data is used, including values for individuals who had their HGS measured at more than one age. Results are weighted. In a first step, simple means (M), standard deviations (SD), and median values (p50) are presented for 14 age groups (S3 Table). Based on OLS regression analysis (with age, height and the variables’ square terms as predictors of HGS), sex-specific *life course profiles* of HGS are estimated and graphically presented. These profiles do not yet account for body height and thus allow for a definition of *peak mean values* for women and men that can be compared with prior studies.

In a second step, age-specific ‘height discount factors’ are calculated based on OLS regressions of HGS on age and body height (and its squared term). To allow for age-specific height stratification, separate regressions are carried out for different age groups (17–24, 25–35, 45–54, 55–64, 65–74, 75–84, 85–90). These regressions are then used to estimate mean HGS across seven height groups (within each of the 14 age groups). The results are presented as sex-specific *reference values*, stratified by age and body height.

Finally, cut-off values are defined to identify persons with a ‘*weak grip’*. Such cut-off values have pragmatically been defined at 2 SD below the *sex-specific peak mean value* in prior research (e.g., [17]). A similar threshold has become a common criterion for the diagnosis of sarcopenia: to allow for an early detection of declining muscle strength, the European Working Group on Sarcopenia in Older Persons (EWGSOP) recommends cut-off points at 2 SD blow the mean value of HGS taken from a healthy and young reference population [2]. To arrive at a more strongly evidence-based risk threshold that relates to a relevant outcome, mortality information from the SOEP is utilized to model survival as a function of HGS. More specifically, these analyses draw on HGS measurements taken in 2006 and mortality information from a follow-up period of 8 years (until the year 2014). HGS measurements are first standardized for age and body height. The standardized measures of HGS are the z-standardized residuals derived from sex-specific OLS regressions of HGS in kg on age (in years) and body height (in cm). Subsequently, the standardized HGS values are recoded into a categorical variable used as the central predictor of mortality, alongside chronological age. The survival analyses (Cox models) are run on a restricted sample of older individuals aged 55–90, i.e., a population group with substantial mortality risks. The sample involves 874 men and 929 women (132 men and 83 women died within the 8-year period).

## Findings

**Fig 1:**
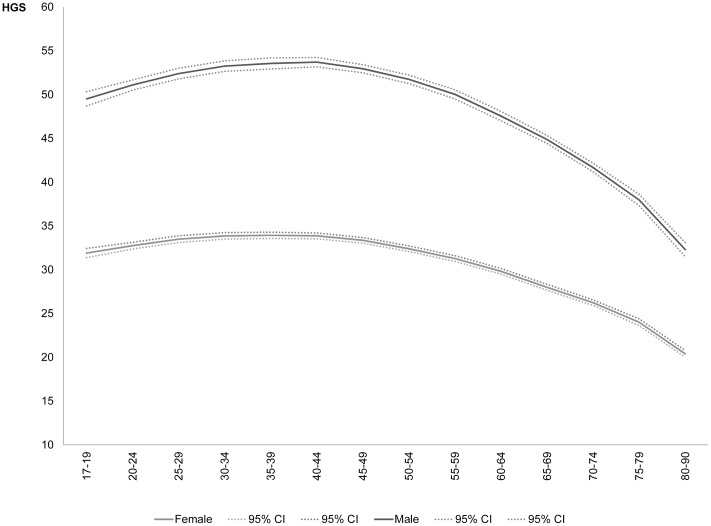
Life course profiles of handgrip strength for German women and men. Predicted values for each age group from a regression of HGS on age, age^2^, height, and height^2^. For simple means and SD by age group, see S3 Table. The graph shows a peak mean value for men of about 54 kg and for women of about 34.5 kg. In the age group 65–69, mean values drop to 44 kg for men and 28 kg for women—values that lie about 1 SD below the peak values.

The estimated *life course profiles* of HGS show a slightly increasing curve up to *peak values* in midlife and a gradual decline in strength thereafter (Fig 1). The mean peak value for men is about 54 kg (ages 30–49), for women it is about 34.5 kg (ages 35–44). Mean values drop to about 44 kg for men and 28 kg for women in the age group 65–69. This drop in HGS between midlife and the second half of the seventh decade of life amounts to almost 1 SD (in the male sample 1 SD pertains to 9.8 kg, in the female sample to 6.8 kg). More information about means, SD, and median values by age group is available from S3 Table. Comparing these results with those presented in recent life course studies (cf. Panel A in S1 Table) suggests that peak values reached in the German population (54 kg for men; 34.5 kg for women) are somewhat higher than those reached in the UK (52 and 31 kg, respectively, see [17]) and the US (49 and 31 kg, respectively, see [19]). Also the values for the seventh decade of life in the German population (44 kg for men; 28 kg for women aged 65–69) remain at a somewhat higher level compared to the UK (42 and 25 kg) and the US (41 and 26 kg). Similar values as the ones presented here are reported by [22] based on a small Bavarian convenience sample.

**Fig 2:**
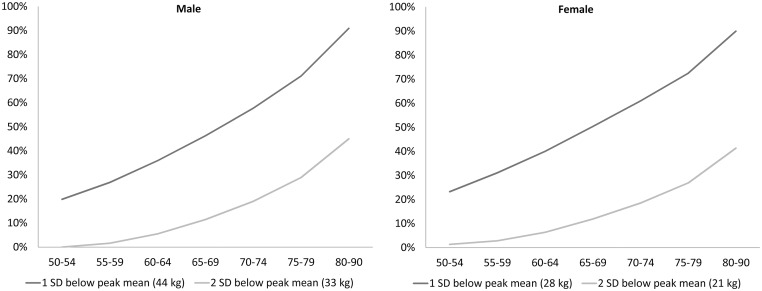
Share of individuals with a *weak grip* using 1 SD and 2 SD cut-offs. The graphs illustrate the rising percentage of men and women with HGS measurements that lie 1 SD or 2SD (weak grip) below the sex-specific peak mean values of about 54 kg for men and 34.5 kg for women. One SD amounts to about 9.8 kg for men and 6.8 kg for women.

The prevalence of a *weak grip*, defined by values that lie 2 SD below the sex-specific peak means (see e.g., [17]), is shown to increase with age (Fig 2). Using this simple approach, a weak grip was defined to start below 33 kg for men and below 21 kg for women. Less than 10% of women and men aged 65–69 are in this way classified as *weak*, shares increasing to 23% of women and 29% of men aged 75–79, and to about half of the population aged 80–90. Interestingly, the prevalence curves of a *weak grip* are very similar for both sexes. Relaxing the definition, using a cut-off at 1 SD below the sex-specific peak mean values, weak grip would be defined to start already below 44 kg for men and 28 kg for women. According to this definition, around 20% of 50–54 year olds would be classified as weak, about half of the population aged 65–69, and about three quarters of the population aged 75–79. The vast majority of octogenarians have a weak grip (88%) according to this alternative definition.

*Normative reference values* for German men and women are shown in Tables 1 and 2. Values are reported for 14 age groups and, within these, for 7 groups defined by body height. For example, the reference value for 40–44 year old women with a body height of 165–169 cm is about 35 kg; this value increases by about 1 kg for every 5 cm of additional height. The relevance of body height for these reference values can be highlighted by the fact that a 20 cm difference in height among women amounts to a similar difference in HGS (of about 4 kg) as comparing women aged 40–44 with those aged 55–59 (i.e., 15 years age difference, keeping height constant). The height effect is even larger among men. The reference values for men aged 40–44 with a body height of 180–184 cm is about 55 kg compared to 52 kg for men who are 10 cm less tall (i.e., about 1.5 kg for every 5 cm of body height). A 20 cm difference in height—comparing 40–44 year old men who are 160–164 cm tall with those who are 180–184 cm tall—amounts to a similar difference in HGS (of about 6.5 kg) as comparing men in their 40s with those in their 60s.

**Table 1:** Normative Reference Values of Handgrip Strength for German Men.

Age	Height	Mean HGS	Risk threshold^1^	Age	Height	Mean HGS	Risk Threshold ^1^
**17–19**	160–164	44.6	34.9	**50–54**	160–164	45.9	37.4
	165–169	45.1	35.4		165–169	47.7	39.2
	170–174	46.5	36.9		170–174	49.4	40.8
	175–179	47.5	37.8		175–179	50.9	42.4
	180–184	48.5	38.8		180–184	52.1	43.6
	185–189	49.3	39.6		185–189	53.1	44.5
	190+	50.4	40.7		190+	54.1	45.5
**20–24**	160–164	47.0	38.8	**55–59**	160–164	42.8	34.3
	165–169	47.8	39.5		165–169	45.1	36.6
	170–174	49.1	40.8		170–174	47.4	38.9
	175–179	50.3	42.0		175–179	49.3	40.9
	180–184	51.2	42.9		180–184	51.1	42.6
	185–189	51.8	43.5		185–189	52.4	43.9
	190+	52.7	44.4		190+	53.5	45.1
**25–29**	160–164	49.4	41.1	**60–64**	160–164	41.0	32.5
	165–169	49.9	41.6		165–169	43.2	34.7
	170–174	50.9	42.6		170–174	45.7	37.3
	175–179	51.9	43.6		175–179	47.6	39.1
	180–184	52.8	44.5		180–184	49.2	40.7
	185–189	54.2	45.9		185–189	50.7	42.2
	190+	56.2	47.9		190+	52.0	43.5
**30–34**	160–164	51.1	42.8	**65–69**	160–164	40.2	32.8
	165–169	51.8	43.5		165–169	42.0	34.6
	170–174	52.6	44.3		170–174	43.6	36.3
	175–179	53.5	45.2		175–179	45.3	37.9
	180–184	54.5	46.2		180–184	46.9	39.6
	185–189	55.9	47.6		185–189	48.9	41.6
	190+	57.3	49.0		190+	50.6	43.2
**35–39**	160–164	47.8	38.0	**70–74**	160–164	37.2	29.6
	165–169	50.2	40.4		165–169	39.1	31.5
	170–174	52.0	42.2		170–174	41.1	33.5
	175–179	53.6	43.8		175–179	42.7	35.2
	180–184	54.9	45.1		180–184	44.4	36.8
	185–189	56.1	46.3		185–189	46.4	38.9
	190+	57.2	47.5		190+	47.6	40.0
**40–44**	160–164	47.9	38.6	**75–79**	160–164	34.7	26.8
	165–169	49.9	40.6		165–169	35.9	28.0
	170–174	51.8	42.5		170–174	37.5	29.6
	175–179	53.4	44.0		175–179	39.0	31.1
	180–184	54.5	45.2		180–184	40.6	32.7
	185–189	55.8	46.4		185–189	42.7	34.8
	190+	56.9	47.6		190+	45.4	37.5
**45–49**	160–164	48.2	39.7	**80–90**	160–164	29.1	21.6
	165–169	50.0	41.5		165–169	31.2	23.6
	170–174	51.6	43.1		170–174	33.0	25.4
	175–179	53.0	44.4		175–179	34.0	26.4
	180–184	54.2	45.7		180–184	35.8	28.3
	185–189	55.4	46.8		185–189	39.5	32.0
	190+	56.4	47.9		190+	40.9	33.3

^1^ group-specific mean value (3^rd^ column) minus 1 age-group-specific SD

**Table 2:** Normative Reference Values of Handgrip Strength for German Women.

Age	Height	Mean HGS	Risk threshold^1^	Age	Height	Mean HGS	Risk Threshold ^1^
**17–19**	150–154	27.8	21.6	**50–54**	150–154	28.2	22.3
	155–159	29.2	22.9		155–159	30.1	24.2
	160–164	30.2	24.0		160–164	31.5	25.6
	165–169	31.2	25.0		165–169	32.9	27.0
	170–174	32.2	26.0		170–174	33.9	28.0
	175–179	33.0	26.7		175–179	35.2	29.3
	180–184	33.8	27.6		180–184	35.6	29.7
**20–24**	150–154	29.1	23.7	**55–59**	150–154	26.9	21.4
	155–159	30.2	24.8		155–159	28.8	23.3
	160–164	31.5	26.1		160–164	30.2	24.7
	165–169	32.5	27.1		165–169	31.2	25.7
	170–174	33.4	28.0		170–174	32.0	26.5
	175–179	34.5	29.0		175–179	32.5	27.0
	180–184	35.0	29.6		180–184	32.9	27.4
**25–29**	150–154	30.8	25.2	**60–64**	150–154	25.8	20.5
	155–159	31.5	25.9		155–159	27.4	22.1
	160–164	32.3	26.7		160–164	28.9	23.6
	165–169	33.3	27.7		165–169	29.9	24.6
	170–174	34.2	28.6		170–174	30.6	25.4
	175–179	35.3	29.7		175–179	31.3	26.0
	180–184	36.4	30.8		180–184	31.5	26.2
**30–34**	150–154	31.4	25.6	**65–69**	150–154	24.5	19.3
	155–159	32.0	26.2		155–159	26.2	21.0
	160–164	32.7	26.9		160–164	27.5	22.3
	165–169	33.7	27.9		165–169	28.6	23.4
	170–174	34.6	28.8		170–174	29.5	24.3
	175–179	35.8	30.0		175–179	30.3	25.1
	180–184	37.0	31.2		180–184	30.5	25.3
**35–39**	150–154	31.0	24.8	**70–74**	150–154	23.4	18.5
	155–159	32.2	26.1		155–159	24.7	19.8
	160–164	33.2	27.0		160–164	26.1	21.2
	165–169	34.3	28.2		165–169	27.3	22.4
	170–174	35.3	29.1		170–174	28.1	23.2
	175–179	36.5	30.3		175–179	28.7	23.8
	180–184	37.6	31.4		180–184	29.2	24.3
**40–44**	150–154	31.5	25.3	**75–79**	150–154	22.7	18.2
	155–159	32.7	26.4		155–159	23.3	18.8
	160–164	33.7	27.4		160–164	24.0	19.5
	165–169	34.8	28.6		165–169	24.9	20.4
	170–174	35.8	29.6		170–174	26.1	21.6
	175–179	37.1	30.8		175–179	27.6	23.1
	180–184	38.0	31.8		180–184	28.9	24.4
**45–49**	150–154	29.8	23.7	**80–90**	150–154	19.9	15.9
	155–159	31.4	25.3		155–159	20.4	16.4
	160–164	32.8	26.7		160–164	21.2	17.1
	165–169	34.1	28.0		165–169	22.1	18.0
	170–174	35.2	29.1		170–174	23.8	19.7
	175–179	36.2	30.1		175–179	23.0	19.0
	180–184	37.0	30.9		180–184		

^1^ group-specific mean value (3^rd^ column) minus 1 age-group-specific SD

The results of the survival analysis (see Table 3 for sample distribution and Table 4 for results) suggest that the mortality risk within 8 years of HGS measurement increases to a significant degree starting with HGS measurements that lie 1–1.5 SD below the group-specific mean value (using an age- and height-standardised measure of HGS). Men whose HGS falls 1–1.5 SD below the *mean value* attained by their peers of the same age and height are found to have a 86% greater hazard to die within 8 years of the HGS test compared to the reference group (HR = 1.9). Also women’s hazard shows a significant increase at 1–1.5 SD below the group-specific mean value of HGS (HR = 2.6). This 1 SD-threshold—which is much lower than the 2 SD cut-off commonly used in gerontological research—is thus used to define a *critically weak handgrip* that warrants closer examination, given the presented evidence for an elevated mortality risk. The estimated group-specific ‘risk thresholds’ in kg are shown in the last columns of Tables 1 and 2. For example, for 65–69 year old men with an average height of 175 cm, mean HGS is estimated at 45 kg and the risk threshold at about 38 kg, whereas for men in the age group 75–79—to take another example—the estimated mean and threshold values (in the same height group) are 39 kg and 31 kg.

**Table 3:** Sample Distributions across Groups Defined by Age- and Height-Standardized HGS.

	N men	N women	% men	% women
Reference group (sM +/- 0.5 SD)	326	388	37.30	41.77
(1) 0.5 SD < 1.0 SD below sM	137	119	15.68	12.81
(2) 1.0 SD < 1.5 SD below sM	78	77	8.92	8.29
(3) 1.5 SD < 3.0 SD below sM	63	68	7.21	7.32
(4) 0.5 SD < 1.0 SD above sM	132	129	15.10	13.89
(5) 1.0 SD < 1.5 SD above sM	75	84	8.58	9.04
(6) 1.5 SD < 3.0 SD above sM	63	64	7.21	6.89
Total	874	929	100.00	100.00

*Notes*: Sample consists of men and women aged 55–90 in 2006, restricted to men with body height 160–200 cm and women with body height 145–185 cm and to those with measured HGS of between 10 and 80 kg. HGS has been standardised for age and height: The *standardised measure of HGS* are the z-standardised residuals (M = 0, SD = 1) from sex-specific OLS regressions of HGS on age and height. Values below -3 SD and above +3 SD are discarded. Abbreviation sM stands for HGS at the *age- and height-standardized mean*.

**Table 4:** Cox Proportional Hazard Models with Age- and Height-Standardized HGS as Categorical Predictor.

	Men		Women	
Predictors	*HR*	*SE*	*HR*	*SE*
Age in yrs	1.09***	0.01	1.11***	0.02
Reference group (sM +/- 0.5 SD)				
(1) 0.5 SD < 1.0 SD below sM	1.41	0.35	1.58	0.53
(2) 1.0 SD < 1.5 SD below sM	1.86*	0.48	2.59**	0.85
(3) 1.5 SD < 3.0 SD below sM	2.05**	0.56	1.58	0.63
(4) 0.5 SD < 1.0 SD above sM	0.39*	0.16	1.13	0.42
(5) 1.0 SD < 1.5 SD above sM	0.55	0.22	0.87	0.39
(6) 1.5 SD < 3.0 SD above sM	0.61	0.26	0.94	0.50
LR chi2(7)	100.57		75.35	
Log likelihood	-828.84		-522.21	
Prob > chi2	0.000		0.000	

*Notes*: Sample consists of men and women aged 55–90 in 2006, mortality follow-up until 2014. N = 874 men (132 deaths) and 929 women (83 deaths). Restriction of sample to men with body height 160–200 cm and to women with body height 145–185 cm. HR: Hazard ratio; SE standard error.

*** p<0.001;

** p>0.01,

* p<0.05

## Discussion

The study presented nationally representative estimates of life course profiles in *handgrip strength* (HGS)–a marker of muscle strength and predictor of future health risks—for Germany. It was based on a large, random sample of the non-institutionalized population. The data at hand provided the rare opportunity to develop normative values for HGS for large parts of the life course (ages 17–90) and the large sample size—compared to most previous work—made it possible to report values stratified by sex, age, *and* body height. The study showed inverted U-shaped life course profiles with mean peak values of about 54 kg for men and 34.5 kg for women and a gradual drop in mean HGS with age from the mid-40s onwards. By age 65–69, average HGS has on average dropped by 1 SD (to 44 kg for men and to 28 kg for women). However, the variance in HGS is substantial. Using a common cut-point in the literature to define a *weak grip* (i.e., 2 SD or more below sex-specific peak mean values), by age 75–79 only about a quarter of the population would be classified as having a *weak grip*. Such pragmatic cut-off values have been widely used in the literature, typically without reference to empirical evidence for the usefulness of this specific threshold in terms of relevant outcomes such as increased risks for cardiovascular disease or mortality. This study used survival analysis to provide such evidence-based thresholds. The findings suggested that the threshold to define *critically weak grip* associated with elevated mortality risks is located already at values that lie 1 SD or more below the standardized mean HGS.

The reference values presented in this study are a valuable source of information in the clinical assessment of HGS and for comparison with studies from other countries. The findings underscore the great importance of accounting for body height when evaluating measured HGS with some reference value.

The presented age-profiles of HGS serve a particular purpose; they are not indicative of ageing processes in terms of muscle strength. Given changes in the health status of populations across cohorts (i.e., older populations from more recent cohorts being less likely to be frail, see Hörder et al. [42], the age-profiles of the provided references values may reflect cohort effects. This would imply that the young of today are likely to show a less pronounced drop in HGS with age than suggested by the profiles (cross-sectional age-profiles of HGS may for this reason overestimate individual decline). Yet, on the other hand, studies on the older parts of any population are subject to selective attrition, less healthy individuals being more likely to drop out due to morbidity and mortality (cross-sectional age-profiles of HGS may for this reason underestimate individual decline). In sum, given potential differences between cross-sectional and longitudinal HGS trajectories, it is important to note that the presented values are for current use as a normative reference for routine measures of HGS in clinical assessment; they not are intended as reference values to monitor individual decline over time.

## Supporting Information

S1 Table Overview of Prior Studies Providing Reference Values for Handgrip Strength. Review restricted to studies published after the year 2000 and excluding studies with very low participant numbers. Values given in pounds (lbs) or Newton have been transformed into (whole) kilogrammes to allow for comparability. *Abbreviations*: D (dominant hand); M (mean values); MD (median values); N (no. of test participants); R (right hand); w/o (without).
(PDF)

S2 Table Overview of Sample Sizes by Survey Year and Sex. Sample: SOEP 2006–2014, HGS test participants, excluding those with HGS < 10 kg, those scoring in the lowest 5% of the PCS, and outliers identified from regressions of HGS on age, age2, height, and height2.
(PDF)

S3 Table Mean and Median Values for HGS by Age, Weighted. Notes: N = 13,120 women; 12,165 men. Table presents weighted means and standard deviations (SD) and median values (P50). The presented values are not standardized for height (for information of mean height by sex and age, see S4 Table. *denotes that the two/four values marked with a * show no statistically significant difference.
(PDF)

S4 Table Mean Body Height in Centimetres by Age, Weighted. Notes: Overall sample size N = 13,120 women; 12,165 men.
(PDF)
