# Supplementary material for: Strong or Weak Handgrip? Normative Reference Values for the German Population across the Life Course Stratified by Sex, Age, and Body Height
Source: PLoS One. 2016 Oct 4;11(10):e0163917. doi: 10.1371/journal.pone.0163917 (PMC5049850; doi:10.1371/journal.pone.0163917)
Supplement: S1 Table — Review restricted to studies published after the year 2000 and excluding studies with very low participant numbers. Values given in pounds (lbs) or Newton have been transformed into (whole) kilogrammes to allow for comparability. Abbreviations: D (dominant hand); M (mean values); MD (median values); N (no. of test participants); R (right hand); w/o (without). (PDF) [file pone.0163917.s001.pdf]

S1 Table. Overview of Prior Studies Providing Reference Values for Handgrip Strength.

Study	Geo	Ages	N participants	Dataset	Measurement	Representativeness	Stratification	Results
Panel A: Life course & nationally representative								
Dodds et al. (2014)	Great Britain	4-94	49,964 participants; 60,803 observations	Values taken from 12 studies incl. *ELSA* and *UKHLS*, measurement in 1990-2012	Varying type of dynamometer, mostly *Jamar*; max. value taken from either hand	Values taken from variety of studies some of which are nationally representative	Sex and 5-year age groups	Peak M men: 52 kg ages 30-39; women: 31 kg ages 25-44<br>Ages 65-69 M men: 42 kg (N=3,947); women: 25 kg (N=4,171)
Peterson & Krishnan (2015)	USA	6-80	7,119 individuals able to perform testing with both hands	National Health and Nutrition Examination Study (*NHANES*) 2011/12	*Takei* digital dynamometer, T.K.K5401; max. value taken from either hand	Yes, for non-institutionalized population	Sex and unevenly spaced age groups	Peak MD men: 51 kg ages 25-30; women: 31 kg ages 20-40 (see Appendix Tab. 2)<br>Age 65 MD men: 42 kg; women: 27 kg (age-specific N unknown)
Perna et al. (2015)	USA	6+ (oldest group 70+)	3,497 males & 3,400 females	National Health and Nutrition Examination Study (*NHANES*) 2011/12	*Takei* digital dynamometer, T.K.K5401; sum of 2 values taken from each hand	Yes, for non-institutionalized population	Sex and unevenly spaced age groups	Peak M men: 49 kg ages 30-39; women: 31 kg ages 30-39 (sum of grip from 2 hands / 2)<br>Ages 60-69 M men: 41 kg (N=373); women: 26 kg (N=354)
Panel B: Life course, not nationally representative								
Peters et al. (2011)	USA	20-96	720 healthy participants (e.g. no ADL restrictions)	Convenience sample recruited from university, hospitals, elderly homes, sports clubs, etc.	*Jamar* dynamometer; max. value taken from either hand	No, convenience sample	Sex, 10-year age groups	Peak MD men: 49 kg ages 40-49; women: 29 kg ages 30-39 (N=50)<br>Ages 60-69 MD men of 43 kg (N=58); women: 25 kg (N=49)
Massy-Westropp et al. (2011)	North West Adelaide, Australia	20+ (oldest group 70+)	1,314 men & 1,315 women w/o hand pain or arthritis	North West Adelaide Health Study	*Jamar* analogue dynamometer	Representative of community-based population of North West Adelaide	Sex and 10-year age groups	Peak M (R) men: 47 kg ages 20-49; women: 31 kg ages 30-39<br>Ages 60-69 M (R) men: 40 kg; women: 24 kg (N not provided)

Schlüssel et al. (2008)	Rio de Janeiro, Brazil	20+ (oldest group 70+)	1,122 men & 1,928 women	Nutrition, Physical Activity and Health Survey (*PNAFS*), 2003	*Jamar*; max. value taken from either hand	Representative of adults living in Niteroi, Rio de Janeiro, Brasil	Sex and 10-year age groups	Peak M (R) men: 47 kg ages 30-39; women: 28 kg ages 30-39<br>Ages 60-69 M (R) men: 37 kg (N=121); women: 22 kg (N=198)
Nilsen et al. (2012)	Norway	20-94	566 participants w/o heart conditions, inflammatory / neurological diseases	Convenience sample recruited from a variety of settings	*Grippit*, electronic instrument, 1 trial with each hand	No, convenience sample	Sex and 10-year age groups	Peak M (R) men: 58 kg ages 30-39; women: 34 kg ages 30-39<br>Ages 60-69 M (R) men 43 kg (N=22); women: 24 kg (N=38)
Günther et al. (2008)	Germany, region of Munich	20-95	403 female & 366 male w/o inflammatory/ neurological disease	Convenience sample recruited from different locations e.g., hospitals, sports clubs, elderly homes	Digital hydraulic dynamometer *NexGen* Ergonomics Inc., mean value from 3 trials per hand	No, convenience sample	Sex and 10-year age groups	Peak M (R) men: 54 kg ages 30-49; women: 33 kg ages 30-39 (N=70)<br>Ages 60-69 M (R) men: 45 kg (N=65); women: 26 kg (N=64)
Werle et al. (2009)	Switzerland	18-96	496 men & 482 women; German-speaking population	Convenience sample recruited in shopping malls, schools, senior residences, etc.	*Jamar* dynamometer, separate values for dominant & non-dominant hand	No, convenience sample	Sex, 5-year age groups	Peak M (D) men: 56 kg ages 35-39; women: 36 kg ages 35-39 (N<50)<br>Ages 65-69 M (D) men: 43 kg(N=46); women: 30 kg (N=34)
Luna-Heredia et al. (2005b)	Spain, 2 cities near Madrid	17-97	287 women & 229 men	Convenience sample recruited in a hospital (patients & visiting relatives)	2 instruments: *Grip-D* & B*ASELINE* dynamometer, max. value from each hand	No, convenience sample	Sex and 10-year age groups	Peak M (D) men: 53 kg ages 30-49; women: 30 kg ages 30-49<br>Ages 60-69 M (D) men: 38 kg (N=41); women: 21 kg (N=24)
Lauretani et al. (2003)	Italy, Chianti region	20-102	469 men & 561 women w/o neurological impairments	*InChianti* epidemiological study	Hand-held dynamometer	Yes, for those living in Greve in Chianti & Bagno a Ripoli	Sex and unevenly spaced age groups	Very small N except ages 65-74:<br>M men: 39 kg (N=230); women: 22 kg (N=255)

Bohannon et al. (2006)	USA, Australia, Canada, UK, Sweden	20+	1,840 women & 1,480 men	Meta-analysis of 12 studies carried out in the 1990s and early 2000s	*Jamar* dynamometer; varying definitions means or max. of several trials	No, collection of convenience samples	Sex, 5-year age groups	Peak M (R) men: 54 kg ages 40-44; women: 34 kg ages 25-34 and 45-49 Ages 60-69 M men: 42 kg (N=202); women: 26 kg (N=250)
Panel C: Age 40+								
Kamide et al. (2015)	Japan	60+	5,216 men & 10,568 women from 97 sets	Meta-analysis of 33 studies, covering several regions	*Smedley*-type dynamometer in 29 studies	Community-dwelling elderly who were independent in ADLs	Sex and 3 specific ages (65, 75, and 85)	Age 65 regression-based estimated M men: 38 kg; women: 24 kg. At age 75 men: 32 kg; women: 21 kg
Yoshimura et al. (2011)	Japan (regional)	40+ (M:71.8)	826 men & 1,642 women, able to walk to test center	*ROAD* study (Research on Osteoarthritis / Osteoporosis Against Disability)	*Toei Light* dynamometer, maximum value of either hand	Population-based cohorts in 3 communities	Sex and 10-year age groups	Ages 60-69 M (D) men: 41 kg (N=137), women: 27 kg (N=316)
Seino et al. (2014)	Japan (regional)	65+ (M: 74.0)	2,097 men & 2,454 women	6 cohort studies collected 2002-2011, Tokyo Metropolitan Institute of Gerontology	*Smedley*-type dynamometer; Max. of 2 trials with D or 1 trial with ND	Nondisabled, community-dwelling adults aged 65+ sampled from 5 regions	Sex and 5-year age groups	Ages 65-69 M men: 35 kg (N=473); women: 23 kg (N=581). Ages 70-74 men: 33 kg (N=699); women 21 kg (N=822)
Ribom et al. (2011)	Sweden (Uppsala)	70-80	999 men, able to walk without aids	*MrOS* study, Uppsala cohort	*Jamar* hydraulic dynamometer, max. of 2 trials in each hand	Random selection of participants from the population registry	Men only, for individual ages between ages 70 and 80	Ages 70-80 M (R) men: 41kg (N=999); at ages 74<76 41 kg (N=191)
Bohannon et al. (2007)	USA, Canada, Australia	75-99	270 men & 469 women	Meta-analysis of 7 studies from 3 countries	*Jamar* dynamometer	No, convenience samples	Sex and 4 age groups (75-79, 80-84, 85-89, 90-99)	Ages 75-79 M (R) men: 33 kg (N=114); women: 22 kg (N=207)
Aoyagi et al. (2001)	Japan & USA	65+	163 Japanese; 9,403 US-Caucasian, 681 US-Japanese women	*Mitsugi Bone & Joint Study; Hawaii Osteoporosis Study; Study of Osteoporotic Fractures*	*Jamar* hydraulic dynamometer, average value of 2 trials	No, regional data from rural farming area in Japan	Women, 5-year age groups, 3 different samples	Ages 65-69 M native Japanese: 25 kg (N=82); US-Japanese: 22 kg (N=112); Caucasians: 23kg (N=4,034)

Panel D: Age 40+ with stratification by body height								
Spruit et al. (2013)	UK	39-73	224,830 (R) persons w/o chronic conditions / obstructive lung function	*UK Biobank*: sample restricted to white ethnic background	*Jamar* hydraulic dynamometer	Yes, for the healthy white population in this age group	Sex, 5-year age groups, 8 height groups	<u>Ages 60<65</u> MD (R) men 170<180cm: 40 kg; women 165<170 cm: 25kg (un-specified large N for sub-groups)
Frederiksen et al. (2006)	Denmark	45-102	8,342 participants; 12,708 observations	3 nationwide population-based surveys, 1998-2003	*Smedley* dynamometer (TTM, Tokio), max. value taken from either hand	Baseline studies were population-representative for particular age groups	Sex, age group, and 7 height groups	<u>Age 65</u> M men: 42 kg if 170 cm tall and 45 kg if 180 cm tall; women: 25 kg if 160 cm tall and 27 kg if 170 cm tall
Kenny et al. (2013)	Ireland	50-85	5,819 participants (size of sub-samples not reported)	*TILDA*, sample w/o cognitive impairment, dementia	B*ASELINE* hydraulic dynamometer; maximum grip from either hand	Yes, for older community dwelling Irish population w/o cognitive impairment	Sex, 5-year age groups, and 2 height groups (for men <173 cm/173+ cm; women <160 cm/160+ cm)	<u>Age 65</u> M men: 34 kg if <173 cm & 38 kg if 173+cm; women 20 kg if <160 cm & 23 kg if 173+ cm. <u>At age 75</u> M men 29 kg if <173 cm & 34 kg if 173+ cm; women 18 kg if <160 cm & 20 kg if 160+cm

Review restricted to studies published after the year 2000 and excluding studies with very low participant numbers (e.g., Mitsionis et al. 2009). Values given in pounds (lbs) or Newton have been transformed into (whole) kilograms to allow for comparability. <u>*Abbreviations*</u>: D (dominant hand); M (mean values); MD (median values); N (no. of test participants); R (right hand), w/o (without)
