# Supplementary material for: Strong or Weak Handgrip? Normative Reference Values for the German Population across the Life Course Stratified by Sex, Age, and Body Height
Source: PLoS One. 2016 Oct 4;11(10):e0163917. doi: 10.1371/journal.pone.0163917 (PMC5049850; doi:10.1371/journal.pone.0163917)
Supplement: S2 Table — Sample: SOEP 2006–2014, HGS test participants, excluding those with HGS < 10 kg, those scoring in the lowest 5% of the PCS, and outliers identified from regressions of HGS on age, age2, height, and height2. (PDF) [file pone.0163917.s002.pdf]

S2 Table. Overview of Sample Sizes by Survey Year and Sex.

	2006	*2008*	*2010*	*2012*	*2014*	*All years*
Age range	17-90	17-90	17-90	17-90	17-90	17-90
HGS range	10.0-79.0	10.5-80.0	10.0-80.0	10.0-80.0	10.0-79.0	10.0-80.0
Height range men	160-200	160-200	160-200	160-200	160-200	160-200
Height range women	150-184	150-184	150-183	150-184	150-184	150-184
N men	2,155	2,286	2,059	2,475	3,190	12,165
N women	2,348	2,405	2,185	2,681	3,501	13,120
N	4,503	4,691	4,244	5,156	6,691	25,285

Sample: SOEP 2006-2014, HGS test participants, excluding those with HGS < 10 kg, those scoring in the lowest 5% of the PCS, and outliers identified from regressions of HGS on age, age2, height, and height2.
