# Supplementary material for: Strong or Weak Handgrip? Normative Reference Values for the German Population across the Life Course Stratified by Sex, Age, and Body Height
Source: PLoS One. 2016 Oct 4;11(10):e0163917. doi: 10.1371/journal.pone.0163917 (PMC5049850; doi:10.1371/journal.pone.0163917)
Supplement: S3 Table — Notes: N = 13,120 women; 12,165 men. Table presents weighted means and standard deviations (SD) and median values (P50). The presented values are not standardized for height (for information of mean height by sex and age, see S4 Table. *denotes that the two/four values marked with a * show no statistically significant difference. (PDF) [file pone.0163917.s003.pdf]

S3 Table. Mean and Median Values for HGS by Age, Weighted.

	Female				Male			
Age	Mean (kg)	SD	P50	N	Mean (kg)	SD	P50	N
17-19	31.5	6.2	31.4	526	48.3	9.6	48.5	538
20-24	32.5	5.4	32.0	799	50.7	8.2	50.5	782
25-29	33.6	5.6	33.5	809	52.4	8.3	52.0	710
30-34	33.3	5.8	33.0	942	53.6*	8.2	53.0	730
35-39	34.2*	6.2	34.0	1144	53.4*	9.8	52.5	924
40-44	**34.5***	6.3	**34.5**	1357	**53.8***	9.3	**54.0**	1,172
45-49	33.4	6.1	33.0	1372	52.9*	8.4	53.0	1,294
50-54	32.2	5.9	32.0	1291	50.4	8.4	50.5	1,189
55-59	30.0	5.5	30.0	1092	49.1	8.5	49.0	1,010
60-64	29.0	5.3	29.0	1004	46.3	8.4	46.5	950
65-69	27.6	5.2	28.0	947	44.1	7.3	44.0	1,019
70-74	26.0	4.9	26.0	895	41.7	7.6	42.0	915
75-79	24.2	4.5	24.0	522	37.8	7.9	38.0	582
80-90	21.4	4.1	21.0	420	33.2	7.5	33.0	350

Notes: N = 13,120 women; 12,165 men. Table presents weighted means and standard deviations (SD) and median values (P50). The presented values are not standardized for height (for information of mean height by sex and age, see Table S4. *denotes that the two/four values marked with a * show no statistically significant difference.
