# Supplementary material for: Strong or Weak Handgrip? Normative Reference Values for the German Population across the Life Course Stratified by Sex, Age, and Body Height
Source: PLoS One. 2016 Oct 4;11(10):e0163917. doi: 10.1371/journal.pone.0163917 (PMC5049850; doi:10.1371/journal.pone.0163917)
Supplement: S4 Table — Notes: Overall sample size N = 13,120 women; 12,165 men. (PDF) [file pone.0163917.s004.pdf]

S4 Table. Mean Body Height in Centimetres by Age, Weighted.

	Female			Male		
Age	Mean (cm)	SD	N	Mean (cm)	SD	N
17-19	167.1	7.4	526	181.1	7.8	538
20-24	167.5	6.2	799	181.3	6.8	782
25-29	167.3	6.5	809	180.5	6.7	710
30-34	167.0	6.9	942	179.9	6.8	730
35-39	166.4	7.4	1144	179.2	7.6	924
40-44	166.7	6.9	1357	179.8	7.4	1172
45-49	166.3	6.7	1372	178.8	7.0	1294
50-54	165.3	6.4	1291	178.0	6.5	1189
55-59	164.6	6.1	1092	177.4	7.0	1010
60-64	163.9	5.7	1004	176.0	6.6	950
65-69	163.1	5.8	947	175.4	5.9	1019
70-74	163.3	5.4	895	174.4	6.0	915
75-79	162.5	4.9	522	173.8	5.8	582
80-90	161.2	4.2	420	172.5	5.2	350

Notes: Overall sample size N = 13,120 women; 12,165 men.
